# Narrowband UVB Phototherapy for Clinically Isolated Syndrome: A Trial to Deliver the Benefits of Vitamin D and Other UVB-Induced Molecules

**DOI:** 10.3389/fimmu.2017.00003

**Published:** 2017-01-24

**Authors:** Prue H. Hart, Robyn M. Lucas, David R. Booth, William M. Carroll, David Nolan, Judith M. Cole, Anderson P. Jones, Allan G. Kermode

**Affiliations:** ^1^Telethon Kids Institute, University of Western Australia, Perth, WA, Australia; ^2^National Centre for Epidemiology and Public Health, Research School of Population Health, Australian National University, Canberra, ACT, Australia; ^3^The Westmead Institute for Medical Research, University of Sydney, Westmead, NSW, Australia; ^4^Centre for Neuromuscular and Neurological Disorders, Western Australian Neuroscience Research Institute, University of Western Australia, Sir Charles Gairdner Hospital, Perth, WA, Australia; ^5^Institute for Immunology and Infectious Disease, Murdoch University, Perth, WA, Australia; ^6^Royal Perth Hospital, Immunology Department, Perth, WA, Australia; ^7^St John of God Dermatology, Subiaco, WA, Australia

**Keywords:** narrowband UVB phototherapy, vitamin D, multiple sclerosis, clinically isolated syndrome, UV-induced immunosuppression, trial, vitamin D supplementation

## Abstract

Low vitamin D and insufficient sun exposure are additive independent risk factors for the development of multiple sclerosis (MS). The usual measure of vitamin D status, serum 25-hydroxy vitamin D [25(OH)D], is also a marker of recent exposure to the UVB rays of sunshine. The main evidence for a protective effect for MS development of higher 25(OH)D comes from observational studies, but this study design cannot separate out whether 25(OH)D is acting as a marker of vitamin D status, sun exposure, or both. In light of a lack of definitive outcomes in MS patients after trials of vitamin D supplementation and the ability of narrowband UVB to induce vitamin D, as well as other immune-regulatory molecules in skin, the Phototherapy for Clinically Isolated Syndrome (PhoCIS) trial was established to investigate the benefits of narrowband UVB, in addition to supplemented vitamin D, on MS development in individuals with Clinically Isolated Syndrome. We propose that the PhoCIS trial provides a fresh approach to re-defining the reported associations of 25(OH)D levels with MS development and progression.

## Introduction

Multiple sclerosis (MS) is a progressive currently incurable, immune-mediated disease of the central nervous system (CNS). Both genetic and environmental factors contribute to MS disease risk, largely through effects on immune pathways [reviewed in Ref. (1, 2)]. Increasing MS incidence, prevalence and mortality with increasing latitude, seasonal effects in relapse rates, and association of MS risk in observational studies with less past sun exposure (1) have led to a focus on insufficient vitamin D as a major risk factor. This was further supported by season of birth effects for MS risk (3). However, studies in humans and mice by us and others [reviewed in Ref. (4, 5)] suggest that immune regulation by UV radiation (UVR) is by both vitamin D-dependent and vitamin D-independent pathways. Our epidemiological work (6, 7) indicates that vitamin D and sun exposure are additive independent risk factors for MS development. A Swedish study has supported these findings (8). Further, direct effects of sun exposure on MRI measures of neurodegeneration in MS, independently of vitamin D, have been reported (9). Thus, associations of 25(OH)D with MS disease progression cannot assume causality of vitamin D and the efficacy of supplementation in the MS disease process even if this is an effective way to raise 25(OH)D levels.

These findings suggest that multiple molecules, including vitamin D, produced in the skin in response to UVR exposure, may contribute to the latitude gradient in incidence, prevalence, and mortality of MS. The question then becomes which strategy might we use to reduce the incidence of MS? Should it be vitamin D supplementation (surely the easiest option with proven safety), or is UVB exposure preferable as it will stimulate the production of vitamin D as well as the multiple other immune-modifying molecules (see below). Here, we introduce the Phototherapy for Clinically Isolated Syndrome (PhoCIS) Trial, which was designed to give individuals at high risk of MS the benefits of both vitamin D and other molecules produced in UVR-exposed skin. Recruitment of participants is ongoing, but here we provide the background and justification for the trial.

## The PhoCIS Trial

The PhoCIS trial examines the effects of narrow band UVB therapy on immune and inflammatory markers of disease, as well as MRI, in patients diagnosed with CIS within the last 120 days (ACTRN12614000185662). The protocol has received approval from the University of Western Australia Human Research Ethics Committee (2014-02-083), and informed consent has been obtained from each participant. All participants receive sufficient vitamin D supplementation to achieve serum 25(OH)D levels of ≥80 nmol/L, as standard care. Half of the participants are randomized to receive the narrowband UVB phototherapy, in addition to vitamin D supplementation. The systemic immune-suppressing effect of UVR exposure has been extensively demonstrated in humans (10, 11). For example, a single sub-erythemal exposure of either 0.25 or 0.5 minimal erythemal dose (the dose of erythemally weighted UVR that causes a just perceptible reddening of the skin) of UVR suppressed contact hypersensitivity responses by 50 and 80%, respectively, of volunteers with Fitzpatrick-defined human skin types I (always burns, never tans) and II (usually burns, tans with difficulty) (12, 13). Human and murine studies suggest a prominent role in UVR-induced immune suppression for dendritic cells (DCs) and induced T-regulatory and B-regulatory cells, culminating in reduced T and B cell responses [reviewed in Ref. (4, 5, 14)].

### Participants

We plan to recruit 60 participants, of which 30 will receive narrowband UVB phototherapy. All participants in PhoCIS have been diagnosed with CIS, the first clinical presentation of a disease that shows characteristics of inflammatory demyelination that could be MS, but has yet to fulfill the criterion of progression in time, i.e., the participants have had only a single clinical episode (15). The primary end point is progression to MS, i.e., a second exacerbation defined by imaging (gadolinium-enhancing lesions or new or unequivocally enlarging T2 lesions) (16). CIS is recognized as a time when the disease course may be more susceptible to intervention resulting in deviation or slowing of disease development. Clinical trials of interferon beta-1a (17) or glatiramer acetate (18) have shown that individuals with CIS who receive treatment are less likely to develop a second exacerbation and have reduced MRI activity. In Australia, CIS patients are not eligible for disease-modifying therapy (DMT), which could complicate assessment of the modulating effects of narrow band UVB phototherapy.

### Intervention

The intervention is very similar to that given to patients with psoriasis where narrow band UVB phototherapy has proven safe and effective, with very minimal adverse side effects (19, 20). Standard phototherapy (UVB between wavelengths 311–312 nm) is given three times/week for 8 weeks (24 exposures in total). Phototherapy is delivered according to the Dundee protocol; a starting dose of 20 mJ/cm^2^ has been chosen based on knowledge of skin type and is well below the threshold for erythema for Fitzpatrick-defined skin types 1–II. All patients start with 40% increments on their initial dose, and this is reduced to 20% increments after six exposures. Using a similar protocol, narrow band UVB exposures were given three times/week for 4 weeks to 33 healthy adults with a mean (±SD) baseline of 25(OH)D of 52.9 ± 10.4 nmol/L (21). Serum 25(OH)D levels increased by a mean of 41.0 nmol/L (95% CI 34.8–47.2) and support claims that narrow band UVB phototherapy will increase vitamin D status. For comparison, 25(OH)D levels increased by 20.2 nmol/L (95% CI 14.6–26.0) from a similar baseline in 30 individuals given 20 µg (800 IU) oral cholecalciferol daily for 4 weeks (21).

### Assessment of Disease Progression

**Figure 1:**
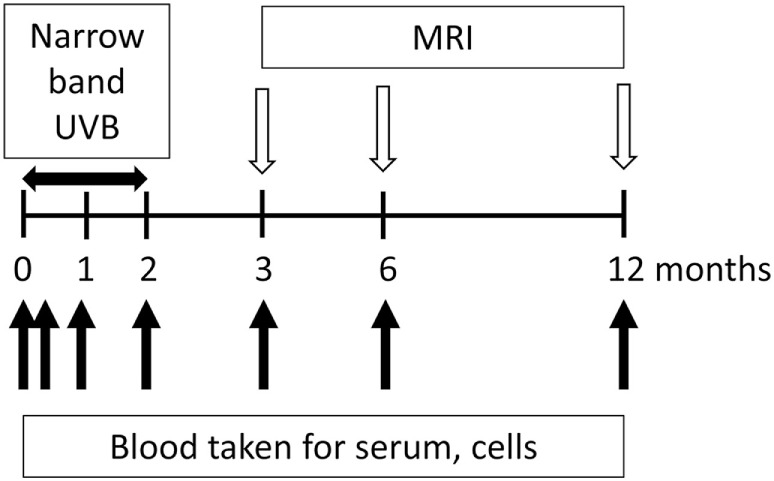
**Flow chart of the study design for Phototherapy for Clinically Isolated Syndrome**. Participants are in the study for 12 months with half given narrowband UVB phototherapy. Blood is collected at the time of enrollment and after 7 days and 1, 2, 3, 6, and 12 months. Narrow band phototherapy is delivered three times per week for the first 8 weeks of their participation in the trial. Progression is monitored by MRIs after 3, 6, and 12 months.

Participants are followed for 12 months, both clinically and with repeated MRI (assessed after 3, 6, and 12 months) (Figure 1) and with assay of standard immunological biomarkers in blood (beyond the scope of this paper). Blood is drawn by venepuncture at the time of enrollment and after the first week, first month, and second month (time of end of phototherapy for those receiving it). Blood is also drawn after 3, 6, and 12 months (Figure 1). We propose that due to the active exchange between blood and the CNS, assessments of the phenotype and function of blood cells will provide a window into changes at the site of the inflammatory, demyelinating lesions associated with development of MS.

The effects of narrowband UVB phototherapy have been previously investigated in nine patients with relapsing-remitting MS, i.e., a more established form of MS [mean time (±SEM) since diagnosis, 13 ± 8 years] (22). All but two participants were on DMT (interferon β 1a, natalizumab, glatiramer acetate). They were given sub-erythemal narrowband UVB phototherapy five times per week for 6 weeks and followed up by MRI and neurological testing for 6 months. Although there were no neurological improvements, the participants felt much better. Serum levels of 25(OH)D increased over the 6 weeks from 35.0 ± 8.0 to 103.7 ± 10.6 nmol/L (mean ± SEM, *n* = 9)(22). Those treated showed an increase in T-regulatory cells and IL-10-producing DCs in UV-irradiated skin and an elevated subset of T-regulatory cells in the blood. When CD4+ cells from the blood were re-stimulated *in vitro*, they expressed less IL-21, which by its function may help to explain the increased T-regulatory cell numbers (23). The significance of these changes in individuals on DMT therapy is under investigation. In PhoCIS, the participants are DMT-free when recruited.

## Why Establish PhoCIS While a Trial of Oral Vitamin D Supplementation Would be Easier?

Supplementation of patients with CIS with oral vitamin D would be simpler and easier to manage. With respect to prevention of MS, there have been two previous studies. The first was a randomized controlled trial in Iran of 30 optic neuritis patients given 50,000 IU vitamin D_3_ per week for 12 months (24). There were significantly fewer cases of a second demyelinating attack measured on MRI in the treated group. The second study was a retrospective case–control study in Norway of self-reported consumption of cod liver oil (5 mL contains 400 IU vitamin D), during childhood, adolescence, and adulthood by MS adults diagnosed for <10 years (25). A significant reduction of risk for cod liver oil consumption was recorded only for intake during adolescence. Thus, there is some but limited evidence of a potential benefit of vitamin D supplementation for the prevention of MS and the two reported studies require verification. In Australia, the PrevANZ study is examining the effects of oral vitamin D supplementation on individuals with CIS with the end point of progression to MS by MRI criteria (ACTRN12612001160120). A similar trial has been established in France (D-Lay-MS, NCT01817166).

The majority of published studies have investigated the treatment of MS with vitamin D; these were recently detailed in a Rapid Response Report by the Canadian Agency for Drugs and Technologies in Health (26). The authors evaluated the clinical effectiveness of vitamin D supplementation in four systematic reviews (27–30), eight randomized controlled trials (24, 31–37), and three non-randomized studies (25, 38, 39). The authors of the CADTH Rapid Report concluded that “the outcomes of vitamin D supplementation for MS were heterogeneous, conflicting and inconsistent, with no effect on disability scores and relapse rates. Further, there were both positive and negative results for immunologic factors, imaging studies, and functional outcomes.” Other studies suggest that ongoing inflammation, as in MS, may hinder systemic increases in 25(OH)D levels with supplementation (40).

## Proposed Mechanism of Prevention of MS Development by UVB Phototherapy: Vitamin D-Dependent Pathways

The precursor of vitamin D in keratinocytes, 7-dehydrocholesterol, absorbs UVB photons to initiate a pathway of vitamin D production, followed by hydroxylations in the liver, kidney, and immune and epithelial cells, and ultimately the biosynthesis of 1,25-dihydroxy vitamin D [1,25(OH)_2_D], the active form of vitamin D (41). Unless supplemented, humans obtain up to 80% of their vitamin D by sun exposure. Many recent reviews have covered the immunomodulatory properties of 1,25(OH)_2_D (42), particularly with reference to regulation of cells proposed important to the development of MS. Genetic variation of the Vitamin D regulating genes, CYP27B1 and CYP24A1, have also been identified as risk factors (2). Many of the properties of 1,25(OH)_2_D have been detected *in vitro* with isolated cell populations and using concentrations of 1,25(OH)_2_D that are much higher than systemic levels, including pharmacological doses. However, immune cells have the enzymes to convert vitamin D or 25(OH)D to 1,25(OH)_2_D, and local concentrations, at the level of the cells, may be much higher than systemic levels (43). Generally, there is evidence that 1,25(OH)_2_D can stimulate the generation of tolerogenic DCs (44), and enhance both the number and function of T-regulatory cells (45). Direct effects on all immune cell types have been reported, which is supported by their expression of the vitamin D receptor. There are also many effects of vitamin D on human immune cells that are still not fully understood, particularly as responses by human and murine cells can vary. For example, 25(OH)_2_D can control T cell antigen receptor signaling and activation of human but not murine T cells (46). Furthermore, trials of vitamin D supplementation for multiple conditions have generally been disappointing (30, 47, 48) and have raised questions about the immunomodulatory abilities of vitamin D *in vivo*.

UV radiation-induced vitamin D may also regulate the development of MS by its effects on VDR-expressing non-immune cells in the CNS. In one study of CIS patients, each 25 nmol/L increase in 25(OH)D level was significantly associated with 7.8 mL higher gray matter volume, and there was a trend for an inverse relationship over 12 months between 25(OH)D levels and new brain lesions and clinical relapses (49). As vitamin D maternal deficiency has been associated with altered development of multiple organs before birth (50), it is possible that vitamin D may positively regulate re-myelination programs.

## Proposed Mechanism of Prevention of MS Development by UVB Phototherapy: Vitamin D-Independent Pathways

Several chromophores in skin for UVB photons have been implicated in vitamin D-independent pathways: *trans*-urocanic acid in the stratum corneum, DNA, RNA, lipids and tryptophan of keratinocytes, and antigen-presenting cells [reviewed in Ref. (4, 5, 10, 11)]. All may initiate pathways involved in signaling from skin to immune cells in draining lymph nodes and tissues beyond. It has been shown that T-regulatory cells are induced locally but then migrate to sites of inflammation, including the CNS, for regulation of experimental autoimmune encephalomyelitis (EAE), the murine model of MS (22). Epidermal Langerhans cells have been implicated in the immune regulation of narrowband UVB phototherapy (51). Importantly, sub-erythemal amounts of UVR, as in narrowband UV phototherapy, can suppress both local and systemic immunity, measured functionally by reduced cell-mediated immune responses. A recent advance in understanding the immune regulation by sub-erythemal UVR has come from studies of keratinocyte-derived host defense peptides, previously called antimicrobial peptides. These peptides have modest antimicrobial activity but rather pleiotropic immunoregulatory properties (52). They are produced in sub-erythemal UV-irradiated skin in the absence of any inflammation (53); they include cathelicidin LL-37 and the α- and β-defensins (52). As shown in a murine model, β-defensins *via* induction of T-regulatory cells can prevent and mitigate EAE (54). Recent studies have also implicated UVR in the maintenance of a pool of B-regulatory cells in the periphery that can prevent an autoimmune attack on the CNS (55). Narrow band UVB irradiation can also increase peripheral T-regulatory cell numbers and their suppressive function in patients with polymorphic light eruption (56).

Further advances to understanding the long-term effects of UVR exposure have come from studies of myeloid progenitors in the bone marrow of UV-irradiated mice (57–59). Immune cells in peripheral organs, including DCs that are the most important cells in initiating immunity, are constantly being replaced by bone marrow-derived hemopoietic cells. We have shown that a single irradiation of skin with erythemal UVR, or multiple sub-erythemal exposures, can suppress contact hypersensitivity responses at distant skin sites for between 1 and 3 months, and suggests bone marrow involvement *per se* (58, 60). Using chimeric mice engrafted with bone marrow from UV-irradiated mice, UV irradiation of skin alters the differentiation program of myeloid progenitors in bone marrow, so terminally differentiated DCs and macrophages are less immunogenic and more regulatory (58, 60). The reduced immunogenicity (confirmed in skin and airways) causes an attenuated ability of bone marrow-derived DCs to respond to inflammatory signals and associated antigens, and to prime new immune responses (58, 60). Adoptive transfer of DCs differentiated from the bone marrow of UV-irradiated mice and loaded *in vitro* with antigen can reduce inflammatory challenge responses in mice already sensitized to that antigen (58, 60), a scenario that can be likened to injected DCs suppressing ongoing autoimmune disease, such as MS. Importantly, the effect of sub-erythemal UVR to skin on bone marrow myeloid progenitors is by a vitamin D-independent, prostaglandin E_2_ (PGE_2_)-dependent process (57), with an epigenetic modification of an early myeloid progenitor in the bone marrow proposed. Involvement of UVR, bone marrow-differentiated DCs, and PGE_2_ in MS pathogenesis is supported by the identification of the gene encoding PTGER4, a PGE_2_ receptor that is an intermediary for the UV effects on DC progenitors in the bone marrow, as an MS risk factor in genome-wide association studies (2). We propose that in patients with CIS, exposure to narrow band UVB will similarly alter the immunogenic properties of circulating DCs and their precursor cells toward a regulatory profile. As UVB exposure can simulate innate immunity (61), we do not anticipate detrimental effects of the narrowband UVB phototherapy on host responses to infection.

## Conclusion

Association of lower 25(OH)D levels with increased risk of MS and greater relapse rate, and the plausibility of 1,25(OH)_2_D as a biochemical modulator of MS pathogenic processes, has encouraged multiple trials of vitamin D supplementation. However, the inconclusive outcomes to date of these trials encourage a fresh approach to re-interpret the reported associations. As 25(OH)D levels are generally a measure of recent sun exposure, we propose that the source of 25(OH)D on MS prevention is investigated. The PhoCIS trial has been established for individuals with CIS who are administered narrowband UVB phototherapy 24 times over 8 weeks and assessed neurologically and immunologically for 12 months. There are multiple immunomodulatory agents, in addition to vitamin D, produced in UVB-irradiated skin; we propose that by giving individuals at high risk of developing MS narrowband UVB phototherapy that they will gain the benefits of both vitamin D and other molecules implicated in UVB-induced immunoregulation.

## Author Contributions

PH, AK, RL, DB, WC, and DN conceived the idea to perform the trial and contributed to trial design. JC is the dermatologist in the team; AJ is the trial co-ordinator. All the authors contributed to drafting the manuscript and have approved the final version of the manuscript.

## Conflict of Interest Statement

The authors declare no competing interests that are of direct relevance to the current research.
